# Epidemiological Predictive Modeling of COVID-19 Infection: Development, Testing, and Implementation on the Population of the Benelux Union

**DOI:** 10.3389/fpubh.2021.727274

**Published:** 2021-10-28

**Authors:** Tijana Šušteršič, Andjela Blagojević, Danijela Cvetković, Aleksandar Cvetković, Ivan Lorencin, Sandi Baressi Šegota, Dragan Milovanović, Dejan Baskić, Zlatan Car, Nenad Filipović

**Affiliations:** ^1^Faculty of Engineering, University of Kragujevac, Kragujevac, Serbia; ^2^Bioengineering Research and Development Center (BioIRC), Kragujevac, Serbia; ^3^Institute for Information Technologies, University of Kragujevac, Kragujevac, Serbia; ^4^Department of Surgery, Faculty of Medical Sciences, University of Kragujevac, Kragujevac, Serbia; ^5^Faculty of Engineering, University of Rijeka, Rijeka, Croatia; ^6^Clinical Centre Kragujevac, Kragujevac, Serbia; ^7^Faculty of Medical Sciences, University of Kragujevac, Kragujevac, Serbia; ^8^Institute of Public Health Kragujevac, Kragujevac, Serbia

**Keywords:** COVID-19, disease spread modeling, SEIRD model, LSTM model, epidemiological model

## Abstract

Since the outbreak of coronavirus disease-2019 (COVID-19), the whole world has taken interest in the mechanisms of its spread and development. Mathematical models have been valuable instruments for the study of the spread and control of infectious diseases. For that purpose, we propose a two-way approach in modeling COVID-19 spread: a susceptible, exposed, infected, recovered, deceased (SEIRD) model based on differential equations and a long short-term memory (LSTM) deep learning model. The SEIRD model is a compartmental epidemiological model with included components: susceptible, exposed, infected, recovered, deceased. In the case of the SEIRD model, official statistical data available online for countries of Belgium, Netherlands, and Luxembourg (Benelux) in the period of March 15 2020 to March 15 2021 were used. Based on them, we have calculated key parameters and forward them to the epidemiological model, which will predict the number of infected, deceased, and recovered people. Results show that the SEIRD model is able to accurately predict several peaks for all the three countries of interest, with very small root mean square error (RMSE), except for the mild cases (maximum RMSE was 240.79 ± 90.556), which can be explained by the fact that no official data were available for mild cases, but this number was derived from other statistics. On the other hand, LSTM represents a special kind of recurrent neural network structure that can comparatively learn long-term temporal dependencies. Results show that LSTM is capable of predicting several peaks based on the position of previous peaks with low values of RMSE. Higher values of RMSE are observed in the number of infected cases in Belgium (RMSE was 535.93) and Netherlands (RMSE was 434.28), and are expected because of thousands of people getting infected per day in those countries. In future studies, we will extend the models to include mobility information, variants of concern, as well as a medical intervention, etc. A prognostic model could help us predict epidemic peaks. In that way, we could react in a timely manner by introducing new or tightening existing measures before the health system is overloaded.

## Introduction

Wuhan was registered on December 19, 2019 as the epicenter of the emergence of a new virus from the group of coronaviruses that showed the characteristics of inter-human transmission, causing a respiratory disease presenting with fever, dry cough, and, often, severe pneumonia with acute respiratory distress syndrome (ARDS) (1). The World Health Organization (WHO) announced the pandemic disease Coronavirus Disease-2019 (2) caused by severe acute respiratory syndrome coronavirus 2 (SARS-CoV-2) and brought measures in order to interrupt the spread of SARS-CoV-2 worldwide. Emerging and recurring diseases have contributed to a renewed interest in infectious diseases. Mathematical models have been valuable instruments for the study of the spread and control of infectious diseases. Understanding the dynamics of transmission of infectious diseases in populations, regions, and countries will contribute to better approaches to reducing the spread of these diseases (3–6). Indeed, the number of infected people grows exponentially, and many countries have decided to impose a complete lockdown of affected cities in order to reduce the number of contacts and stop the spread of the virus (7).

To this end, several approaches have been suggested to predict the spread of COVID-19 in population. Compared with standard statistical methods (8, 9), mathematical models based on dynamic equations (10–12) attract comparatively less consideration, although they may provide more insight into the dynamics of epidemics (11). Modeling of infectious diseases is most conveniently performed using deterministic compartmental models. Adjusting the parameters of the equations allows better modeling of environmental characteristics, such as social restrictions (7). These models are based on flow patterns between compartments such as susceptible (S), exposed (E), infected (I), and recovered (R); therefore, their names are often referred to as SEIR, SIT, SIRS, etc. Among all these models, the classical susceptible, expose, infectious, recovered (SEIR) model is the most commonly used concept for characterizing the epidemic of COVID-19 in both China and other countries (11). On the basis of the SEIR model, the success of different interventions after the epidemic can be assessed (13–16), which seems to be a daunting challenge for general statistical techniques. One widely used model is the SIR (susceptible, infected, recovered) model for human-to-human transmission, which defines the migration of persons through three mutually incompatible periods of infection: prone, contaminated, and recovered (6). However, Yi-Cheng et al. assert that the traditional SIR model neglects time-varying properties, such as transmission and recovery rate (17). They discuss that the time-independent SIR model is too simple to precisely and effectively predict the trend of the disease. Therefore, they suggested a time-dependent SIR model, where both rates are functions of time *t*. Also, several models have been developed for the COVID-19 pandemic. Lin et al. have developed the SEIR (susceptible, released, infectious, deceased) model taking into account some parameter estimates from the 1918 influenza pandemic (18). Anastassopoulou et al. suggested a discrete SIR model with deceased individuals (19), Casella developed a control-oriented SIR model that highlights the consequences of delays and measures the results of various containment policies (20), and Wu et al. used propagation dynamics to measure the clinical magnitude of COVID-19 (21). Stochastic transmission models were also considered (22, 23). Giordano et al. suggest a new epidemiological mean-field approach for the COVID-19 outbreak in Italy, expanding the classic SIR model, close to that developed by Gumel et al. for SARS (24). The mentioned expanded SIR model, named SIDARTHE, considers eight stages of infection: susceptible, infected, diagnosed, ailing, recognized, threatened, healed, and deceased (6). The SIDARTHE model recognizes a difference among infected individuals depending on whether they have been diagnosed, and on the severity of their symptoms. The difference between diagnosed and non-diagnosed individuals is important, because the ones who have been diagnosed as positive are isolated, and it is less probable they will spread the infection. This delineation also helps to explain the incorrect interpretation of the epidemic spread and the case fatality rate (6).

In addition to the aforementioned models and variations, there are attempts in the literature to build agent-based models (ABMs) with various purposes and goals. Indeed, ABMs have long been used to simulate various illnesses (25, 26). As a result, ABMs have grown in popularity for modeling the spread of COVID-19 and analyzing alternative approaches to the problem (27–29). Notably, several studies have investigated the effects of contact tracing on the transmission of COVID-19 (30–33). Two most distinct and complete software in this are COVID-ABS and Covasim. Silva et al. (34) developed a methodology for COVID-ABS, a novel SEIR (susceptible-exposed-infected-recovered) ABM that seeks to mimic pandemic dynamics by simulating individuals, businesses, and governments using a society of agents (34). Seven different scenarios of social distancing interventions were investigated, each with a different epidemiological and economic impact: (1) do nothing, (2) lockdown, (3) conditional lockdown, (4) vertical isolation, (5) partial isolation, (6) use of face masks, and (7) use of face masks in conjunction with 50% adhesion to social isolation. Kerr et al. (32) developed a methodology of Covasim (COVID-19 agent-based simulator) (35). Covasim, among others, supports a broad range of interventions, namely, non-pharmaceutical interventions like physical separation and protective equipment, pharmaceutical interventions like vaccination, and testing interventions like symptomatic and asymptomatic testing, isolation, contact tracing, and quarantine. Delays, loss-to-follow-up, micro-targeting, and other variables can all be incorporated into these treatments. A use case was presented for Seattle/King County, Washington, United States from January 27 to November 14, 2020, with projections until December 31, including additional restrictions imposed on November 16. Its complexity proved to be adequate to examine epidemic dynamics and inform policy decisions.

Since epidemiological measures do not always give the expected and desired results and a pandemic is constantly changing its course, it is obvious that a new approach is necessary to improve the existing measures to fight the epidemic. Deep learning methods, such as recurrent neural networks (RNNs), are well-suited for modeling temporal sequences (36). However, the main limitation of RNNs is reflected in learning of long-term dependencies in large sequences that can involve hundreds or thousands of steps. These limitations are addressed by long short-term memory (LSTM) networks (37). There are also several studies that investigate LSTM as an approach to forecasting future COVID-19 cases (38–42). LSTM was used for COVID-19 forecasting in Canada and achieved an accuracy of 93.4% for short-term and 92.67% for long-term predictions (39). For COVID-19 forecasting in China, LSTM was also used, and in comparison with the dynamic SEIR model, LSTM achieved promising results (43). Results showed LSTM achieved good forecasting performance because of its capacity to handle time-dependent datasets. Ismail et al. modeled data from Denmark, Belgium, Germany, France, the United Kingdom, Finland, Switzerland, and Turkey using LSTM among several methods. They stated that LSTM is the most accurate model in comparison with the two other investigated algorithms, and they provided LSTM in order to make predictions in a 14-day perspective (44). This model is able to make realistic estimates based on the current situation and predict accurately the number of confirmed and recovered cases. Chandra et al. compared RNNs, LSTM networks, bidirectional LSTM networks, encoder-decoder LSTM networks, and convolutional neural networks (CNNs) with focus on univariate time series for multi-step-ahead prediction. The results showed that the encoder-decoder LSTM network, in addition to bidirectional LSTM, provides the best performances for given time series problems (37).

By predicting and preventing epidemiological peaks, we could achieve a “flattened curve” of the spread of the disease in order to prevent such a rapid spread of disease that could lead to overloading of national health systems and their collapse (45). Owing to the scarcity of the official data available and many unknown parameters in COVID-19 epidemic spread, development, and control, most early published models were prone to over-fitting, or parameters were taken from literature/on the basis of restricted and less precise evidence. This results in ambiguous results, especially because many articles are published ahead of peer review. Therefore, the main objectives of this study are to:

- carefully collect, harmonize, and unify epidemiological data from reliable sources, state, regional, and local levels, and incorporate them into the proposed models,
- investigate and compare two approaches in modeling the COVID-19 epidemiology, the SEIRD model and the deep learning model based on LSTM networks,
- train and test the investigated models on the cases in Benelux countries.

## Materials and Methods

We propose two main approaches, a standard compartmental epidemiological SEIRD model and a deep learning model based on LSTM networks, to describe the spread of COVID-19. From the aspects of disease progression notations, it should be emphasized that notation “severe cases” is equivalent to “hospitalized cases,” and “critical cases” is equivalent to “ICU cases” in our article. However, for the training of the LSTM model, we have used the original data with the terms infected, hospitalized, and ICU; and for the SEIRD model, taking into account its definition, we have used subsequently derived terms mild, severe, and critical. The term deceased is a common term for both models.

The processing hardware included 64 GB of RAM, an NVIDIA Quadro RTX 6000 GPU, and an Intel(R) Xeon(R) Gold, 6240R, CPU running at 2.40 GHz. Tensorflow and Keras were used to implement the network in the Python 3.7.4, using the Spyder 3.3.6 environment.

### Compartmental SEIRD Epidemiological Model

**Figure 1:**
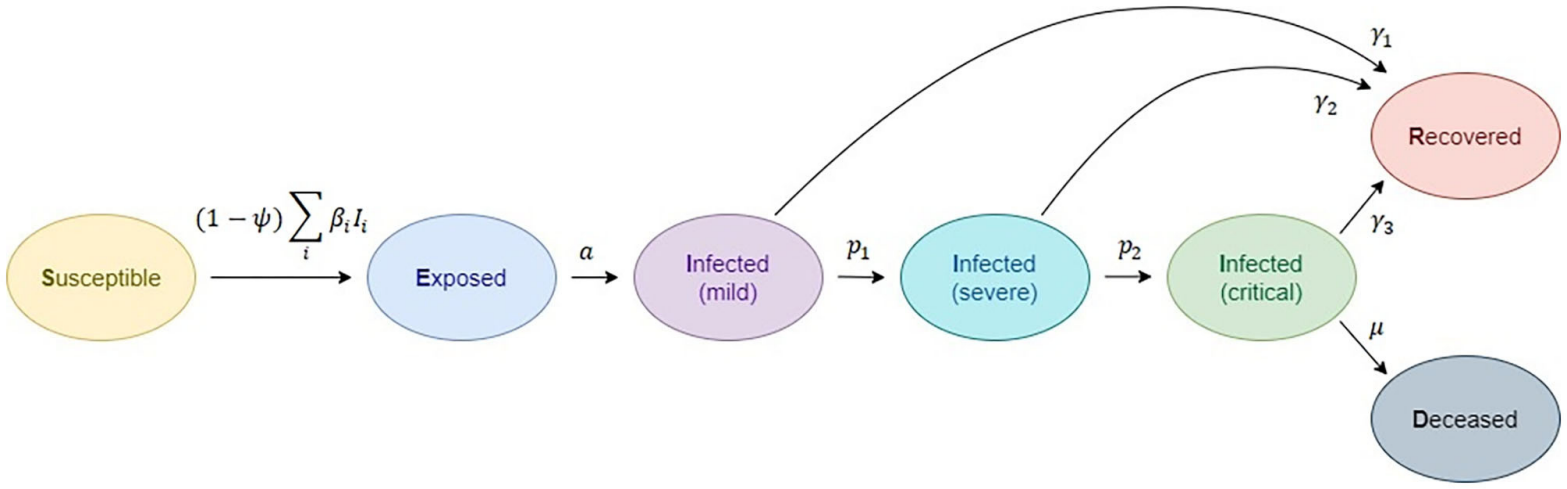
Compartmental epidemiological model, based on the classic susceptible, exposed, infected (1- mild, 2-severe, 3-critical), recovered, deceased (SEIRD) model.

The basic structure of the SEIRD model is inspired by a number of studies on the natural clinical progression of COVID-19 infection (46). The choice for compartments to be used in the model depends on the features of the individual disease being modeled and the intent of the model. Passively immune class M and latent period class E are often ignored, because they are not essential to susceptible-infective experiences (3). The SEIRD model should more accurately reflect the progression of the epidemic than the simpler SIRD model that does not include an incubation period. The model used in this study is given in Figure 1. Although the standard SEIRD model has been used, to our knowledge, no previous models have included the division of infected class into three subclasses, mild, severe, and critical. Proposed SEIRD models, therefore, use a standard definition of compartments with the extension of modeling three infection stages. In addition, we have extended our model with a transmission rate mitigation factor to simulate the introduction of a variety of social measures (lockdown, etc.), mainly referring to the methodology introduced by Bastos et al. (47) and Morato et al. (48).

This yields to differential equations for the definition of disease spread:


(1) $$\begin{array}{l} Ṡ=-\left(1-\psi \right)\cdot \left(\beta_{1}I_{1}+\beta_{2}I_{2}+\beta_{3}I_{3}\right)S \end{array}$$

where *S* represents the susceptible individuals who are infected. We have adapted the classical SEIRD model to include the effect of isolation by including ψ(*t*), which represents a transmission rate mitigation factor. This factor expresses the observed social isolation ratio within the susceptible population. This means that ψ = 0 is the case where there is no control over viral load (no social measures introduced), while ψ = 1 represents a complete lockdown, without any social interactions (47).

Disease spread in the exposed (E) community is further described as


(2) $$\begin{array}{l} Ė=\left(1-\psi \right)\cdot \left(\beta_{1}I_{1}+\beta_{2}I_{2}+\beta_{3}I_{3}\right)S-aE \end{array}$$

The rate of development from the exposed stage to the infected stage *I*, where the patient becomes symptomatic and contagious, occurs at a pace *a*. Medical details of the various phases of infection are given below. Infected individuals begin with mild infection (*I*_1_),


(3) $$\begin{array}{l} \overset{∙}{I_{1}}=aE-\left(\gamma_{1}{+p}_{1}\right)I_{1} \end{array}$$

from which they either recover, at the rate of γ_1_ or advance to severe infection (*I*_2_) at the rate of *p*_1_:


(4) $$\begin{array}{l} \overset{∙}{I_{2}}=p_{1}I_{1}-\left(\gamma_{2}{+p}_{2}\right)I_{2} \end{array}$$

Severe infection subjects recover at rate γ_2_ or progresses to critical stage (*I*_3_) at *p*_2_ rate:


(5) $$\begin{array}{l} \overset{∙}{I_{3}}=p_{2}I_{2}-\left(\gamma_{3}+\mu \right)I_{3} \end{array}$$

Recovered persons are defined by *R* class/compartment and are supposed to be safe from re-infection for life:


(6) $$\begin{array}{l} Ṙ=\gamma_{1}I_{1}+\gamma_{2}I_{2}+\gamma_{3}I_{3} \end{array}$$

Individuals with critical infection recover at a rate of γ_3_ and die at a rate of μ.


(7) $$\begin{array}{l} Ḋ=\mu I_{3} \end{array}$$

Individuals can transmit the infection at any point, although at different levels. The transmission rate at stage *i* is defined by β_1_. This means that the used notation is: *S*: susceptible individuals; *E*: exposed individuals, infected but not yet infectious or symptomatic; *I*_*i*_: infected individuals in severity class i, where severity increases with the increase of i, and we assume individuals must pass through all previous classes; *I*_1_: mild infection; *I*_2_: severe infection; *I*_3_: critical infection; *R*: individuals who have recovered from disease and are now immune; *D*: deceased individuals. Total population size is assumed to be constant in the form:


(8) $$\begin{array}{l} N=S+E+I_{1}+I_{2}+I_{3}+R+D \end{array}$$

In order to describe the rates of disease progression from one category to another (susceptible to infected, infected to recovered or deceased, etc.), we use the following notation:

- β_*i*_ rate at which infected individuals in class *I*_*i*_ contact susceptible and infect them,

- *a* rate of progression from the exposed to infected class,
- γ_*i*_ rate at which infected individuals in class *I*_*i*_ recover from disease and become immune,
- *p*_*i*_ rate at which infected individuals in class *I*_*i*_ progress to class *I*_*i*+1_ and
- μ death rate for individuals in the critical condition.

The effects of lockdowns are, therefore, introduced, but due to the complexity of modeling that includes heterogeneity of policies in lockdown and mobility contacts, we have decided to decompose the modeling into shorter periods with respect to one consistent lockdown measure, and perform independent modeling on each of the sub-periods. In this study, we have adopted the value of ψ based on the social measures of each of the investigated countries.

For Belgium (49), on March 12^t^, 2020, the Belgian federal government announced multiple far-reaching measures to flatten the curve of COVID-19 cases. The first measures included obligatory weekend closures of restaurants, bars, and nightclubs, as well as any non-essential stores until the end of the month. Schools at all levels were forced to close as well. Belgium went into its first lockdown on March 17. Non-essential shops had to close completely, working from home became the normal method of operation, and any non-essential movement or travel was forbidden. On March 20, national borders were mostly blocked, and most border traffic was halted. Despite the decision of the government to implement an exit plan, i.e., gradually loosening the restrictive restrictions, the measures remained in effect until the beginning of May. Expectedly, infection rates rose again, and by the end of July, the proclamation of new measures became urgent. From October 16 onward, restrictions were introduced again. A second full-fledged lockdown was in place until the end of November. Based on this, we have defined a table of time-dependent factor ψ(*t*) (Table 1). We have also incorporated a delay effect on the social distancing measures. The values are adopted based on the definition of finitely parametrized social distancing measures defined by Morato et al. (48). We stress that values of transmission rate mitigation factor should be taken only as guidelines that do not affect the conceptual essence of this study, since the proposed methodology is general and can be applied with respect to the epidemic reality of any location and population.

**Table 1:** Adopted values for transmission rate mitigation factor based on social measures (Belgium).

**Time period**	**ψ(*t*)**
Before March 17th 2020	0
March 17th–May 31st 2020	0.9
June 1st−31st July2020	0.7
July 31st–October 16th 2020	0.4
October 16th–December 31st 2020	0.8
December 31st 2020–March 15th 2021	0.7

For Netherlands, after the introduction of lockdown at the end of March, there was a visible decrease in the number of infected people after this period. Strict measures were extended until May 3, and public events were banned until August. Measures were loosened at the beginning of August, resulting in another peak (although smaller than the first one, that was a result of no measures). This peak was also correctly predicted using the SEIRD model. It should be emphasized that the model is able to predict both increase and decrease in the number of cases, however, we outline here only the first two predicted peaks, as peaks are of greatest concern. Results on RMSE are given in Table 4, taking into account modeling for the whole period March 15, 2020 to March 15, 2021.

In Luxembourg, the measures of lockdown were introduced similarly as in Belgium, and the first peak in the epidemic was a result of previous viral load. Lockdown easing and protective measures were gradually loosened with the opening of cinemas and intra-European borders form June 15. This resulted in another peak, which was correctly predicted using the SEIRD model. It should be emphasized that the model is able to predict both increase and decrease in the number of cases; however, we outline here only the first two predicted peaks, as peaks are of greatest concern. RMSE is given in Table 4, taking into account modeling for the whole period March 15, 2020 to March 15, 2021.

Time variables in the proposed model are adopted from the literature:

- Average incubation period (in days),
- Average duration of mild infections (in days),
- Average duration of hospitalization (time to recovery) for individuals with severe infection (in days),
- Average duration of ICU admission (until death or recovery) (in days).

It should be mentioned that the average duration of hospitalization and those of infective periods do not include the incubation period, meaning that the total number of days from exposure to recovery should be the sum of the incubation period and average duration of hospitalization. The aforementioned values of COVID-19 parameters adopted from the literature are given in Table 2.

**Table 2:** Adopted parameters for COVID-19 clinical progression based on literature sources.

**Parameter name**	**Adopted literature value**	**References**
Average incubation period, days	5–6 days; 5 days	(1, 2, 50–56)
Average duration of mild infections, days	7–12 days; 5 days	(50, 57)
Average fraction of (symptomatic) infections that are mild	80	(50, 58, 59)
Average fraction of (symptomatic) infections that are severe^a^	15	(50, 58)
Average fraction of (symptomatic) infections that are critical^a^	5	(50, 58)
Case fatality rate (fraction of infections that eventually result in death)^a^	2.27%	(1, 60, 61)
Average duration of hospitalization (time to recovery) for individuals with severe infection, days	5–14; 10 days	(50, 59, 62)
Average duration of ICU admission (until death or recovery), days	14 days; 12–17 days	(50, 59, 62)
Reproduction number*^b^*	2–2.5; 2	(50, 63)
Infective period mild infection (in days)	5 days	(50, 57, 61, 64)
Infective period severe infection (in days)	7–12 days	(50, 57, 65, 66)
Infective period critical infection (in days)	14 days	(50, 57, 59, 61)
Transmission rate of mild infections	0.4 per day	As a default we assume that the dominant source of transmission comesfrom individuals with mild infections (e.g., β_1_ > β_2_ > β_3_), who are likely to still be in the community, as opposed to isolated in the hospital.
Transmission rate of severe infections	0.2 per day	
Transmission rate of critical infections	0.14 per day	

a *Parameters marked with this footnote are calculated based on available official data, and we did not use the literature data stated in Table 1. Values used are given in these table*.

b *The threshold for many epidemiological models is the specific reproduction number,R0, which is defined as the average number of secondary infections created when an infectious organism is introduced into a host population where everyone is susceptible. In many deterministic epidemiological models, infection will begin in a completely susceptible population only if an R0 > 1 is present*.

When it comes to the evaluation of model coefficients that are compatible with current clinical evidence, we primarily calculated certain parameters from real data available for the countries of Benelux [Belgium (67), the Netherlands (68), and Luxembourg (69)]. We focus on these countries because of the fact that the data were available in a tabular format with division into infection categories (ICU patients, hospitalized patients etc.). Not many countries provide data in such a format to the public. Available data for these countries that were important for calculating the parameters are: the number of infected patients, the number of hospitalized patients, the number of patients on ventilators, and the number of deaths. From these data, it is possible to calculate fraction of infections that eventually result in death, given as


(9) $$\begin{array}{l} case\;fatality\;rate=\frac{Total\;deceased\;cases}{Total\;infected\;cases} \end{array}$$

In order to account for the delay with respect to the duration from the onset of infection to death, we have used adapted CFR (case fatality rate) based on definition from Shim (70). The denominator of the crude CFR formula includes infected persons whose fates are unknown, and those who have not yet died from the disease but will do so in the future. As a result of the time lag between infection and death, the CFR is skewed, a phenomenon known as right censoring (71). Statistical methods have been used to estimate the delay-adjusted CFR (70). The factor of adjustment, *u*_*t*_, has been defined as


(10) ut=∑i=0t∑j=0∞ci-fjj∑i=0tci

where *u*_*t*_ represents the underestimation of the known outcomes and is used to scale the value of the cumulative number of cases in the denominator in the calculation of the CFR, *c*_*t*_ is the daily case incidence at time *t*, and *f*_*t*_ is the proportion of cases with a delay of *t* from onset to death.

Also, it is possible to calculate the average fraction of infections that are mild, severe, and critical. People who are infected but not hospitalized have a mild infection defined as


(11) $$\begin{array}{l} mild\;infections=1-\frac{Total\;hospitalized\;cases}{Total\;infected\;cases} \end{array}$$

where total hospitalized cases and total infected cases were taken from official reported data. Similar to adapting the CFR, delay was incorporated in the formulae given in equations 11–13. Therefore, in equations 11–13, we also include the time of symptom onset to diagnosis, time of symptom onset to hospitalization, as well as hospitalization stay. Overall, the delay between symptom onset and hospitalization can be described by a truncated Weibull distribution with a shape parameter 0.845 and a scale parameter 5.506. The methodology was based on (72). Severe infection refers to individuals who are hospitalized but not on ventilators, and critical infection refers to those on ventilators, both of which are defined as


(12) $$\begin{array}{r} severe\;infections\\ =\frac{Total\;hospitalized\;cases-total\;cases\;on\;ventilators}{Total\;infected\;cases} \end{array}$$

(13) $$\begin{array}{l} critical\;infections=\frac{Total\;cases\;on\;ventilators}{Total\;infected\;cases} \end{array}$$

Based on the described methodology, parameters calculated for Belgium, Netherlands, and Luxembourg are given in Table 3.

**Table 3:** Estimated parameters for COVID-19 clinical progression based on real data.

**Parameter name**	**Estimated value**		
	**Belgium (%)**	**Netherlands**	**Luxembourg**
Average fraction of (symptomatic) infections that are mild	95.96	96.89	98.65
Average fraction of (symptomatic) infections that are severe	4.04	3.11	1.35
Average fraction of (symptomatic) infections that are critical	0.86	1.05	0.2

### LSTM Epidemiological Model

Since the course of the epidemic is rapidly changing and the mathematical SEIRD models have some limitations due to the nature of partial differential equations, we implemented an algorithm that is suitable for data fitting and forecasting based on time-series data. For the problems of this category, adequate architecture will be based on recurrent neural networks (RNNs). The specificity of RNNs is reflected in the context layer, whose purpose is to act as memory in order to merge the current state and inputs for the propagation of information into latter states. A method for training RNNs is backpropagation through time (BPTT), which is a kind of extension of the backpropagation algorithm. BPTT is characterized by a gradient descent where the error is backpropagated for a deeper network architecture that contains time-defined states. For this reason, there is the problem of learning long-term dependencies, which will lead to vanishing and exploding gradients. In order to address the vanishing gradient problem of RNNs, long short-term memory (LSTM) neural network was developed. LSTM is a special kind of recurrent neural network structure that can comparatively learn the proposed long-term dependencies and overcome the mentioned drawbacks of RNNs. LSTM contains memory cells and gates that provide better capabilities in remembering the long-term dependencies. An LSTM cell was introduced by Hochreiter and Schmidhuber (73). Compared with the RNN memory cell, the LSTM memory cell has two components to its state: the hidden state and the internal cell state.

**Figure 2:**
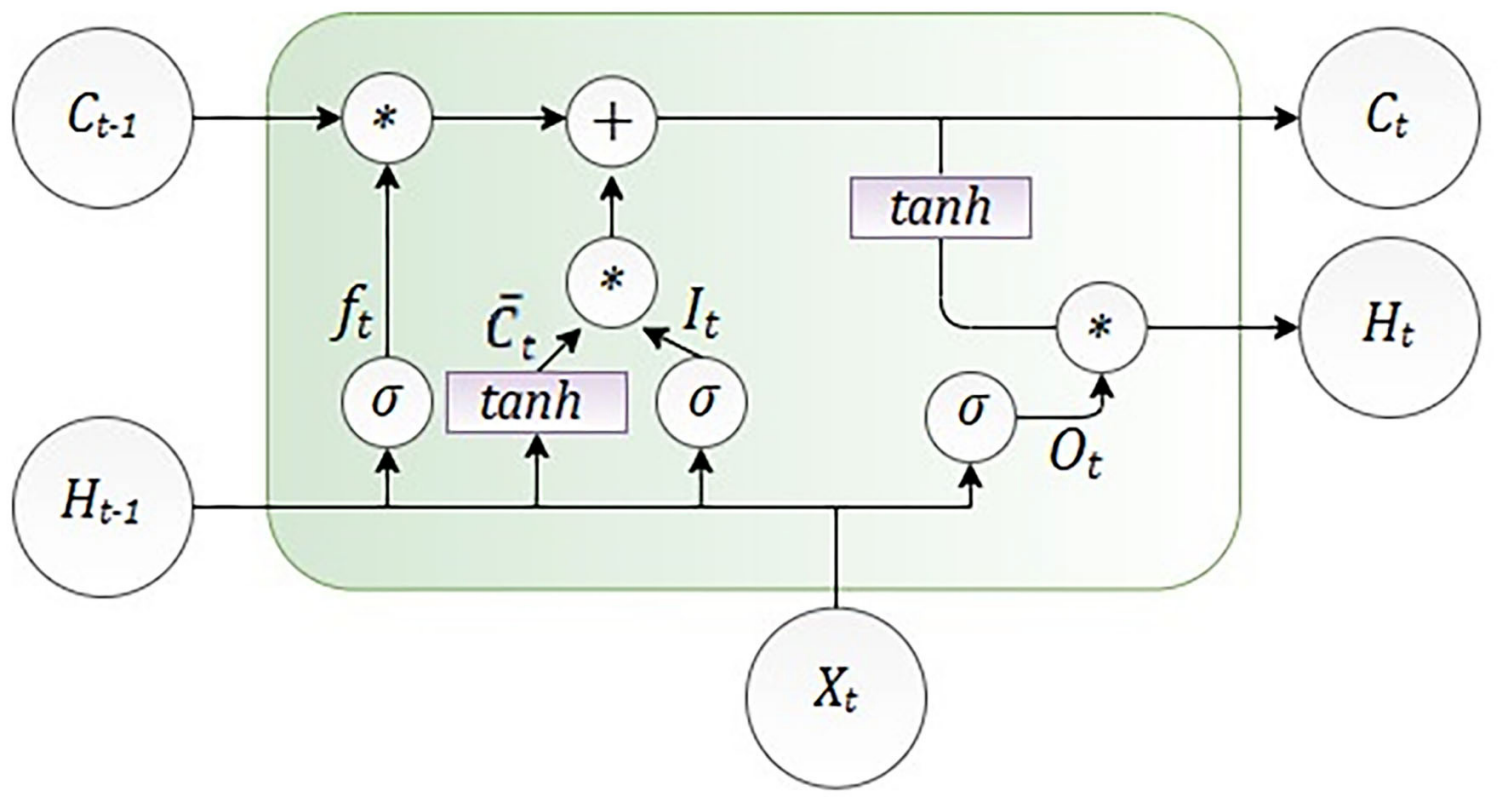
Architecture of one long short-term memory (LSTM) cell.

The LSTM cell consists of the gates shown in Figure 2, the input gate *I*_*t*_ decides which information can be transferred to the cell, then forgets gate *f*_*t*_ decides which information from the previously cell should be neglected. The control gate ${\bar{C}}_{t}$ is controlling controls the update of the cell, and the output gate *O*_*t*_ controls the flow of output activation information.

As it is shown in Figure 2, LSTM calculates hidden layer *H*_*t*_ as


(14) $$\begin{array}{l} I_{t}=\sigma \left(W^{i}\times \left(X_{t}+H_{t-1}\right)+b_{i}\right) \end{array}$$

(15) $$\begin{array}{l} f_{t}=\sigma \left(W^{f}\times \left(X_{t}+H_{t-1}\right)+b_{f}\right) \end{array}$$

(16) $$\begin{array}{l} O_{t}=\sigma \left(W^{o}\times \left(X_{t}+H_{t-1}\right)+b_{o}\right) \end{array}$$

(17) $$\begin{array}{l} {\bar{C}}_{t}=\tanh\left(W^{c}\times \left(X_{t}+H_{t-1}\right)+b_{C}\right) \end{array}$$

(18) $$\begin{array}{l} C_{t}=\sigma \left(f_{t}*C_{t-1}+I_{t}*{\bar{C}}_{t}\right) \end{array}$$

(19) $$\begin{array}{l} H_{t}=\tanh\left(C_{t}\right)*O_{t} \end{array}$$

The number of the input features is presented as *X*_*t*_, and *H*_*t*_ is the number of hidden units. Learning started with the zero initial values of *C*_0_ and *H*_0_. Also, during the learning process, some parameters were adjusted, such as bias given as *b* and weight given as *W*. The internal memory of the unit is given as *C*_*t*_, and it should be emphasized that all the gates have the same dimension as the size of your hidden state (74).

**Figure 3:**
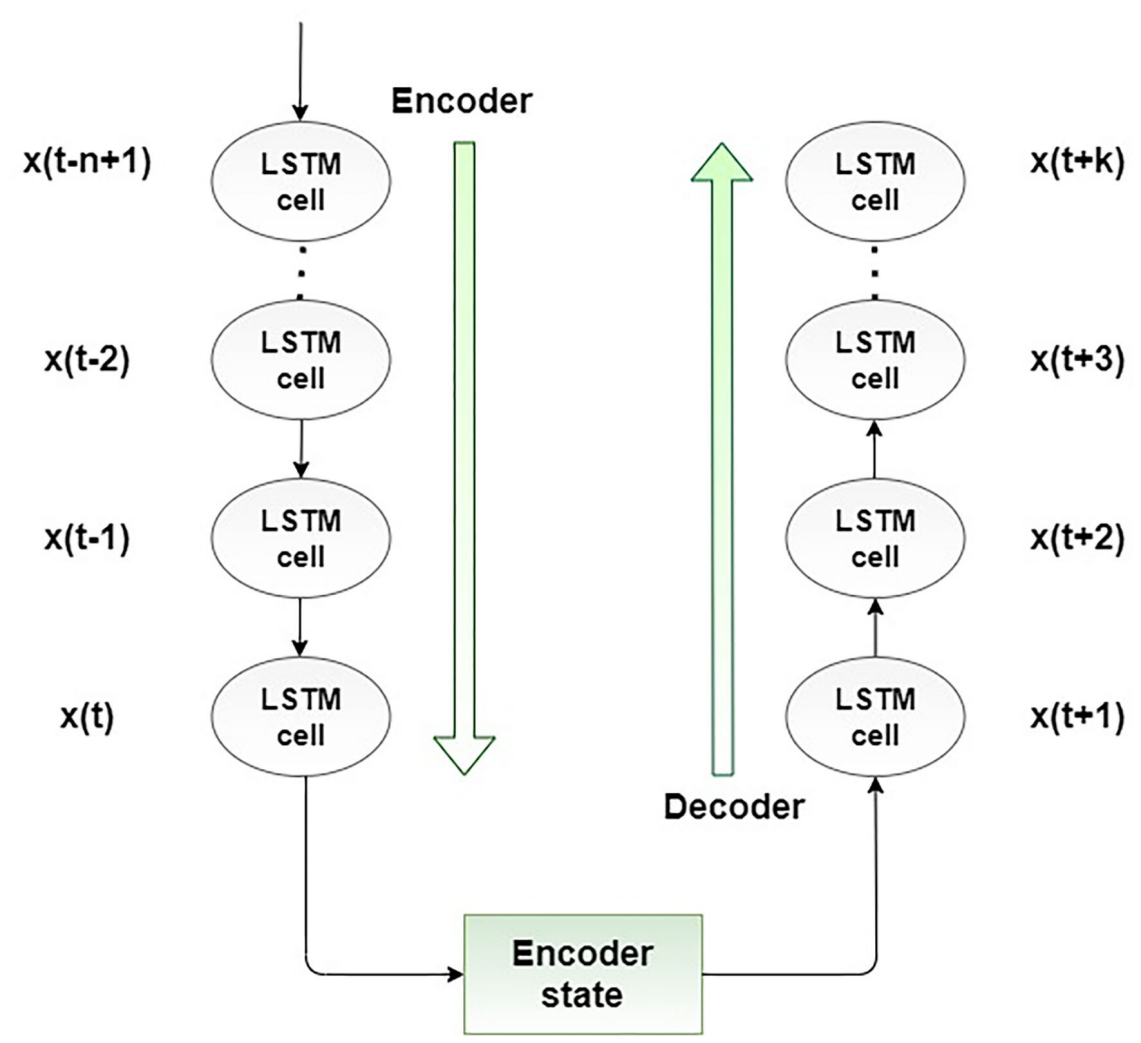
Proposed encoder-decoder LSTM structure.

The encoder-decoder LSTM (ED-LSTM) network was developed as a sequence-to-sequence neural network to effectively map a fixed-length input to a fixed-length output. The advantage of these neural networks is that the mentioned two lengths of inputs and outputs do not have to be the same. For that reason, this neural network achieved state-of-the-art results in the field of automatic text translation. The ED-LSTM network has two implementation phases: the first phase, encoding, and the second phase, decoding. The purpose of the first phase is to encode an input sequence into a fixed-length vector representation and compute a sequence of hidden states, and the purpose of the second phase is to decode the vector representation and define a distribution of the output sequence. The architecture of the ED-LSTM is presented in Figure 3.

## Results and Discussion

In this section, we present the results of the applied SEIRD model and LSTM encoder-decoder model, and compare them with the real situation of the COVID-19 outbreak and spread in Benelux countries. Data were monitored during the 1-year period, from March 15, 2020 to March 15, 2021. Values for the numbers of people infected, hospitalized, in ICU as well as deceased were taken from Infectious Diseases Data Explorations & Visualizations (68) for Belgium (67) for Netherlands, and (69) for Luxembourg.

### Results for the SEIRD Model

The results for the SERID model showed that the model is effective at predicting several peaks of the epidemiology. Due to the fact that transmission rate mitigation factor was included in the model to simulate the social measures (no isolation to complete lockdown), the model is able to catch more than one peak, but also captures the fall in the trend of epidemiological situation. It should be emphasized that predicted numbers represent current numbers (daily predictions) of the infected cases (mild, severe, critical), but the number of deceased cases is cumulative.

**Figure 4:**
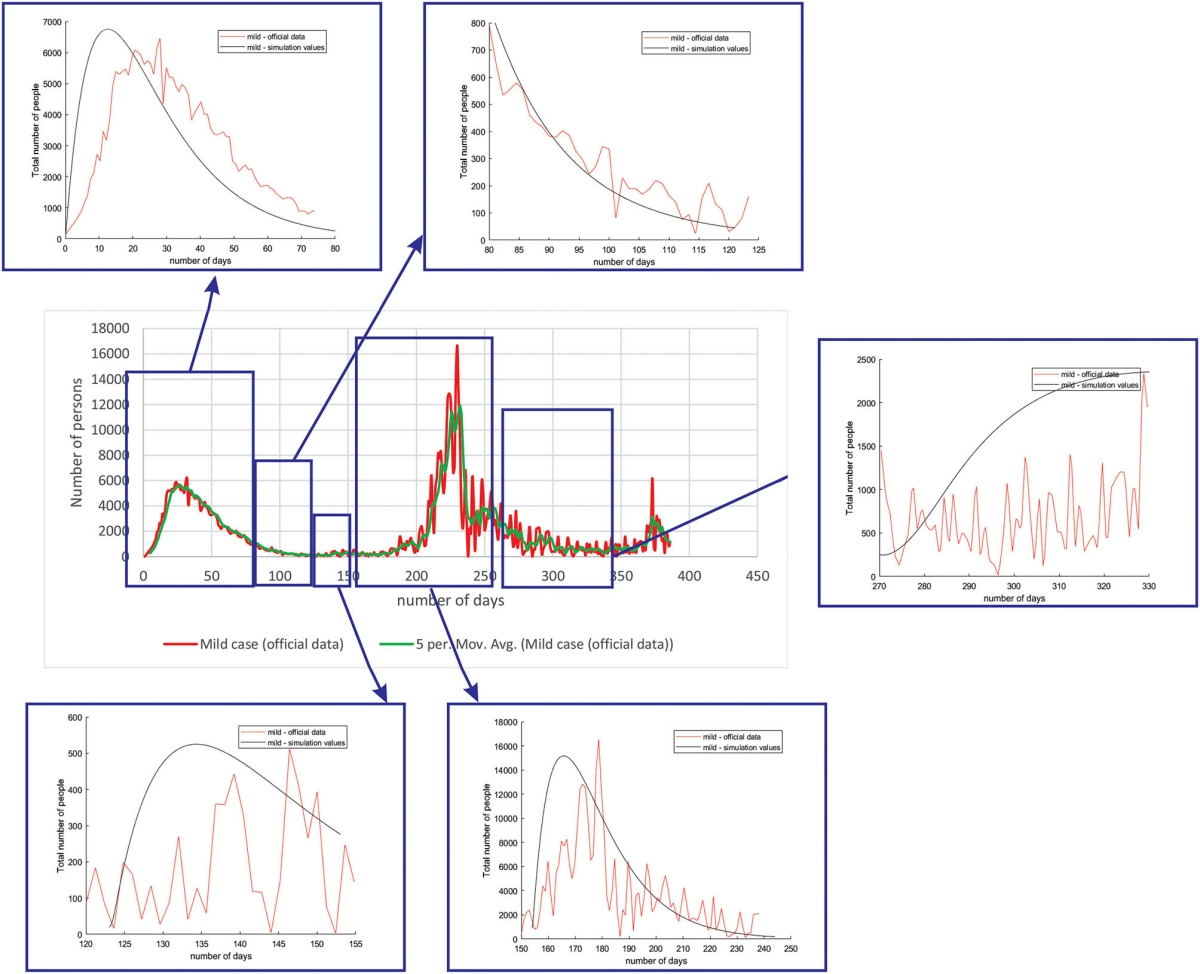
Comparison between the real and simulated values for mild cases in Belgium using the proposed SEIRD model.

Figure 4 shows the comparison between the official data and simulated curves for mild cases in the aforementioned characteristic sub-periods. The match between the trends of the real epidemiological situation and predicted progress shows that the model is able to follow the course of epidemiology, whether there is an increase or decrease in the number of infections.

**Figure 5:**
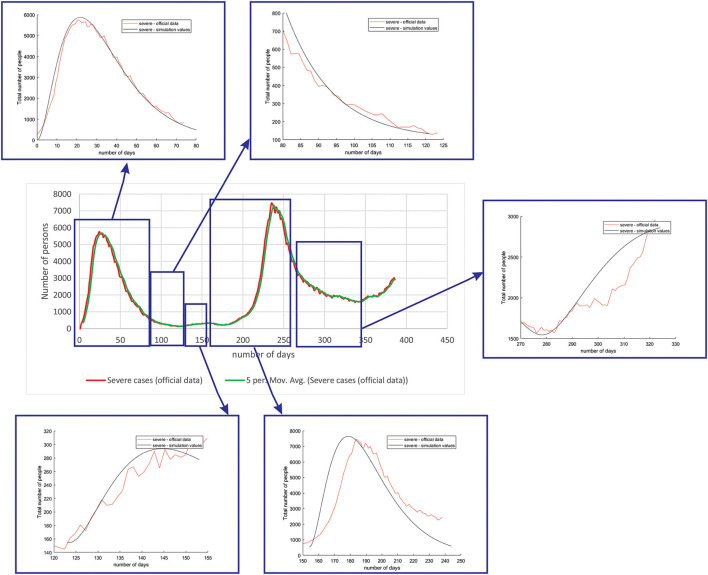
Comparison between the real and simulated values for severe cases in Belgium using the proposed SEIRD model.

Figure 5 shows the comparison between the official data and simulated curves for severe cases in the aforementioned characteristic sub-periods. The match between the trends of the real epidemiological situation and predicted progress here is even better than for the mild cases.

**Figure 6:**
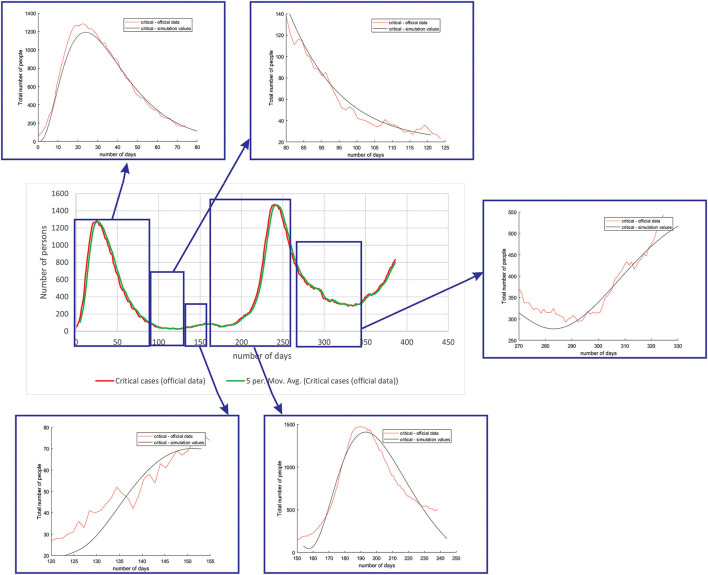
Comparison between the real and simulated values for critical cases in Belgium using the proposed SEIRD model.

Figure 6 shows the comparison between the official data and simulated curves for critical cases in the aforementioned characteristic sub-periods. The difference between the trends and peak positions of the real epidemiological situation and predicted values is small, with an almost perfect fit.

**Figure 7:**
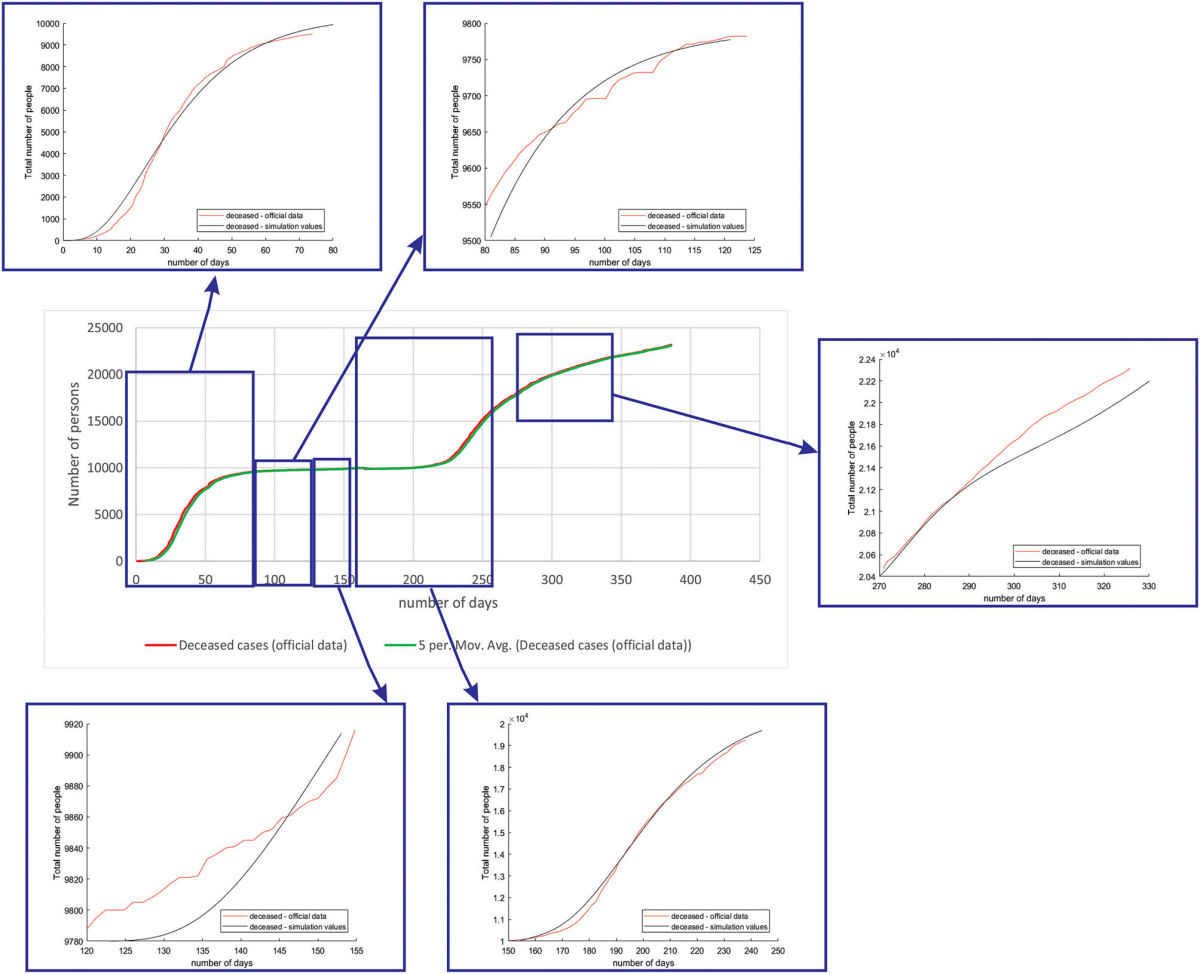
Comparison between the real and simulated values for deceased cases in Belgium using the proposed SEIRD model.

Figure 7 shows the comparison between the official data and simulated curves for deceased cases in the aforementioned characteristic sub-periods. The difference between the trends and peak positions of the real epidemiological situation and predicted deceased values is small, with an almost perfect fit.

As it can be seen, tracking the number of people with severe infection, critical infection, or deceased people shows a very good match. The trend and peak value of predicted curves show a promising match for all the monitored curves, mild, severe, critical, and deceased. Some differences can be observed in peak positions for some sub-periods between the simulated and real cases. This is may be due to the initial conditions set in simulation, or adopted values of certain parameters from literature, which can be further optimized. Nevertheless, the trend is adequate, showing that the methodology can be used to describe the epidemiology.

The same methodology was performed for all the three countries (Belgium, Netherlands, and Luxembourg), as well as investigated groups (mild, severe, critical, and deceased). We only present the complete figures for Belgium to demonstrate the methodology, while for Netherlands and Luxembourg, we only show the match for peaks, as they are of greatest interest.

**Figure 8:**
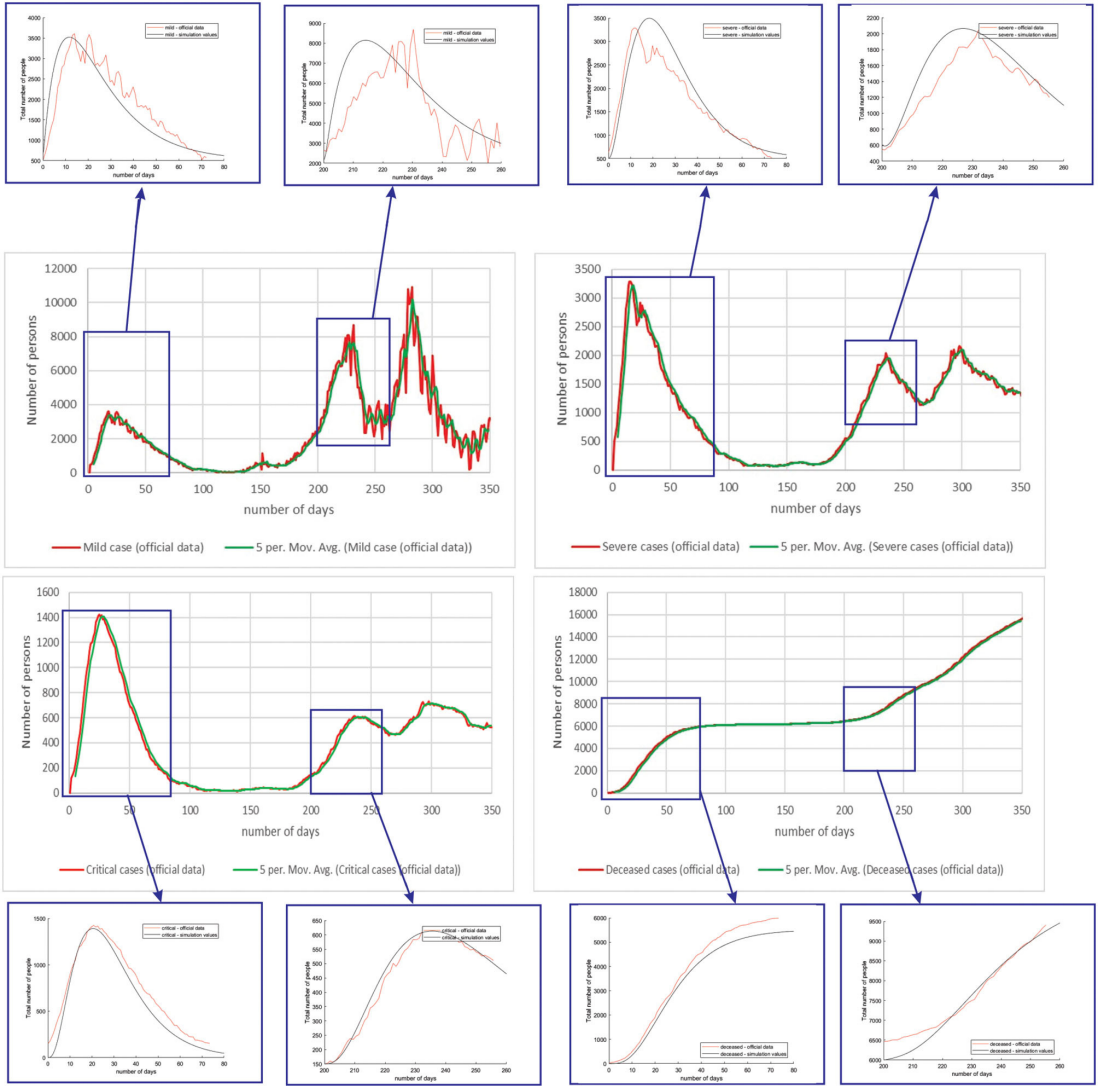
Comparison of real and simulated values for the four categories of interest (Netherlands), mild infection, severe infection, critical infection, and deceased people.

Figure 8 shows the comparison between the official reported data and simulated curves. Regarding the comparison of official statistical and simulated values, for the case of Netherlands, the trend in all four classes, infected critical, infected severe, infected mild, and deceased are matching, as well as the position of the peaks. It can be seen that in the cases of infected people with a severe or critical condition, as well as deceased people, the curves match perfectly with very small deviations between simulated and official data.

**Figure 9:**
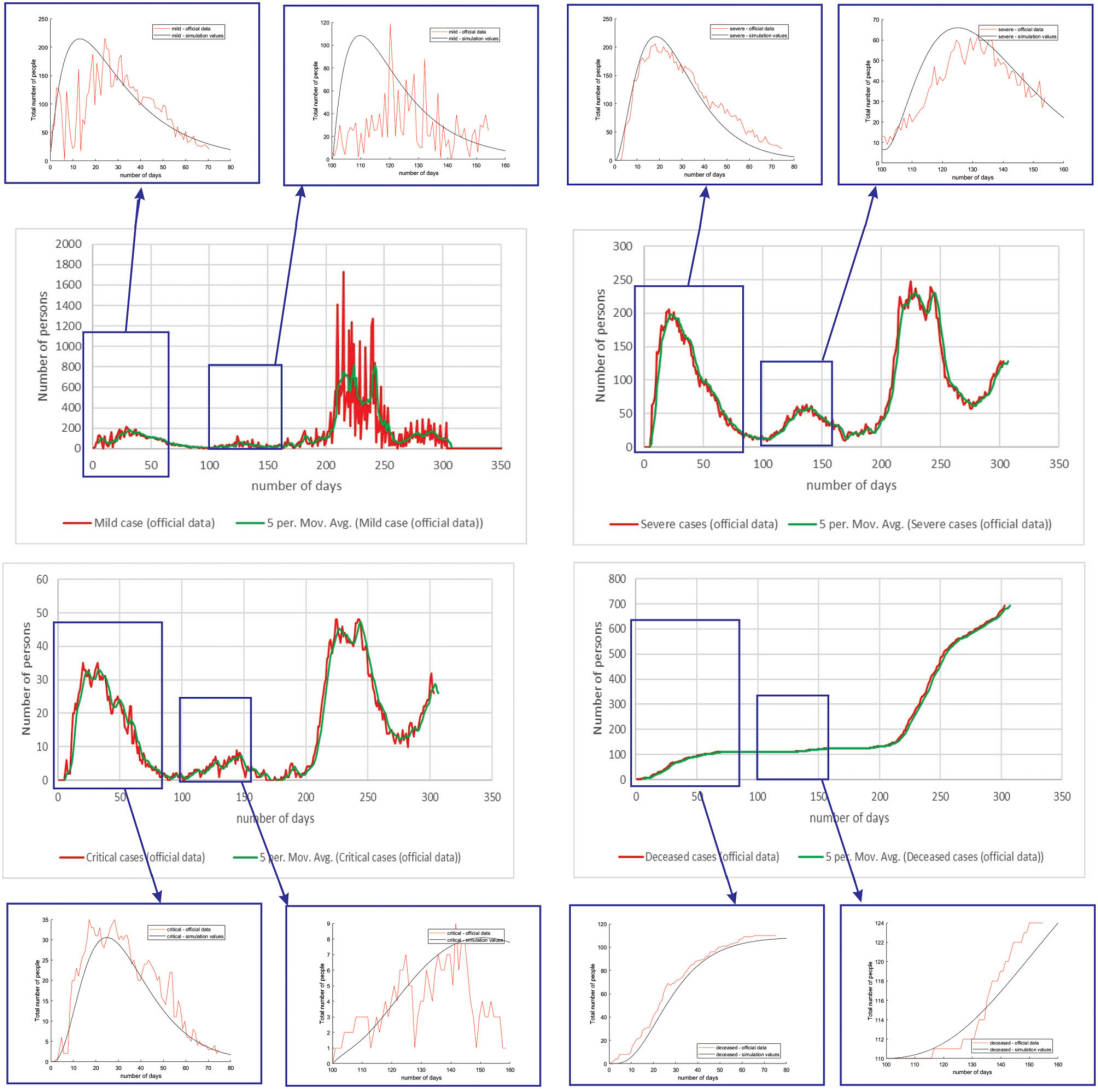
Comparison of real and simulated values for the four categories of interest (Luxembourg), mild infection, severe infection, critical infection, and deceased people.

For the case of Luxembourg, it can be seen that the trend and fluctuations are matching for number of simulated and real number of people with mild infections. For classes severe and critical infections, as well as deceased, the trends and values of the predicted simulation line match well with the official reports (Figure 9).

Table 4 shows the root mean square error (RMSE) between simulated and real values of investigated curves in time for Belgium, Netherlands, and Luxembourg. The values are given as mean ± standard deviation, because of the fact that we have calculated RMSE for each modeled subperiod. We applied the moving average smoothing technique to the real values in order to remove the fluctuations between days and calculated RMSE between the smoothed curve and simulated curve. The results show very small RMSE for all three countries and classes of infection. The only larger value of RMSE is for mild infections, which can lie in the fact that mild cases were derived based on other data (total cases and hospitalized cases), as there are no available data on specific mild category.

**Table 4:** Root mean square error between simulated and official statistical data for Belgium, Netherlands and Luxembourg using SEIRD model.

**RMSE**	**Belgium**	**Netherlands**	**Luxembourg**
	**mean** **±** **std**	**mean** **±** **std**	**mean** **±** **std**
Mild infected	240.79 ± 90.556	219.52 ± 20.49	198.54 ± 78.53
Severe infected (hospitalized)	7.47 ± 2.64	2.49 ± 0.1	2.24 ± 0.83
Critical infected (ICU)	0.58 ± 0.49	0.05 ± 0.002	0.15 ± 0.1
deceased	49.09 ± 40.82	21.7 ± 6.4	4.98 ± 2.11

All this shows that there are many factors involved in the prediction of the epidemic spread, and that more factors can be taken into account in order to account for decreasing and increasing trends, etc. However, if looking at shorter periods of time and modeling on such subperiods, number of deceased and infected people (mild, severe, and critical), using official statistics and simulated numbers shows good match, which means that the models is showing promising results and can be further upgraded to take into account different underlying complex phenomena.

In comparison with the existing literature, Bastos et al. (47) proposed an adapted susceptible-infected-recovered (SIR) model, for the purpose of incorporating under-reporting in Brazil and the response of the population to confinement measures, widespread use of masks, etc. They discuss that the most optimal method for epidemiological modeling should be based on recurrent model tweaking (through identification), with the uncertainty margins always taken into consideration (47). Morato et al. (48) also investigated modeling using the SIRD model, which includes time-varying auto-regressive immunological parameters in the case of Brazil (48). Their main contribution is adding analytical regressions, least-squares optimization, and auto-regressive model fits. Köhler et al. (75) studied adaptive techniques for resilient and optimum management of the COVID-19 pandemic using social distancing measures and applied them in the case of Germany (75). Using the SIDARTHE model, they introduced key features that distinguish between detected and undetected cases, symptomatic and asymptomatic individuals, with the additional separate state for patients with life-threatening symptoms. Similarly, in the case of Germany, Kantner et al. (76) proposed adjustments to traditional SEIR models by introducing the optimal solution computed by minimizing the terminal cost function. Alleman et al. proposed a discrete model predictive controller for optimal government response to the COVID-19 outbreak that would not result in overloading the number of ICU beds in Belgium (77). Their method mostly focused on calibration of the social interaction parameter, which results in an improved model in comparison with the state-of-the art models.

Our model introduces a traditional SEIRD model but with improved methodology, classification of infected class into three subclasses, mild, severe, and critical. It also takes into account the transmission rate mitigation factor as a form of modeling social measures (partial to full lockdown etc.). Time-related parameters are considered with time delays. RMSE is competitive with presented state-of the results, with space for additional improvements. The added value of this study lies in the validation of the proposed methodology using official data from national authorities in Benelux countries. Despite the fact that some aspects of epidemics are taken into account, there are certain simplifications of the model. The main limitations of the SEIRD model may come from different aspects:

- values of parameters estimated based on official statistics are not correct, and official statistics are scarce or not reported accurately: it has already been reported in other articles that official statistics have underreported the real numbers in the beginning, leading to the fact that initial conditions were not taken correctly and, therefore, later simulated, and real numbers were different. A predictive model by Imai et al. used travel volumes from Wuhan and used the dates when imported cases first arrived in cities within China to forecast the size of the epidemic in Wuhan (78). They reported that substantially more cases were present in Wuhan than were reported in the official statistics (79). The same conclusion was met in an article by Korolev (80). He asserts that even though the fraction of all cases that are reported are not identified, it can be effective to consider the underreporting. If it is assumed that all cases are reported and the estimates of R_0_ are based only on that, the value of R_0_ may be biased downward. It may lead to overestimating the number of deaths (80).
- the complexity of the COVID-19 epidemic spread and development is yet to be determined, and the current SEIRD model does not take into account medical interventions, number of hospital beds available, etc. The real case is much more complex, with many other additional phenomena included (behavioral responses to the epidemic, re-infection, no immunization, mutations of the virus, variants of concern, mobility of people, etc.)

Although the model is showing promising results, and the match between the curves between the simulated trends and values calculated based on officially available data are well-matching, there are some differences in peak values and positions. This can primarily be due to the fact that modeling the spread of the disease is complex and includes many phenomena, out of which only several main are included in the current model. Therefore, the main limitation of this study is limited number of phenomena modeled (no reinfection, asymptomatic infection, medical intervention, etc.). Therefore, we have investigated models based on deep learning and further report the results for one such LSTM-based model.

### Results for the LSTM Encoder-Decoder Neural Network

**Figure 10:**
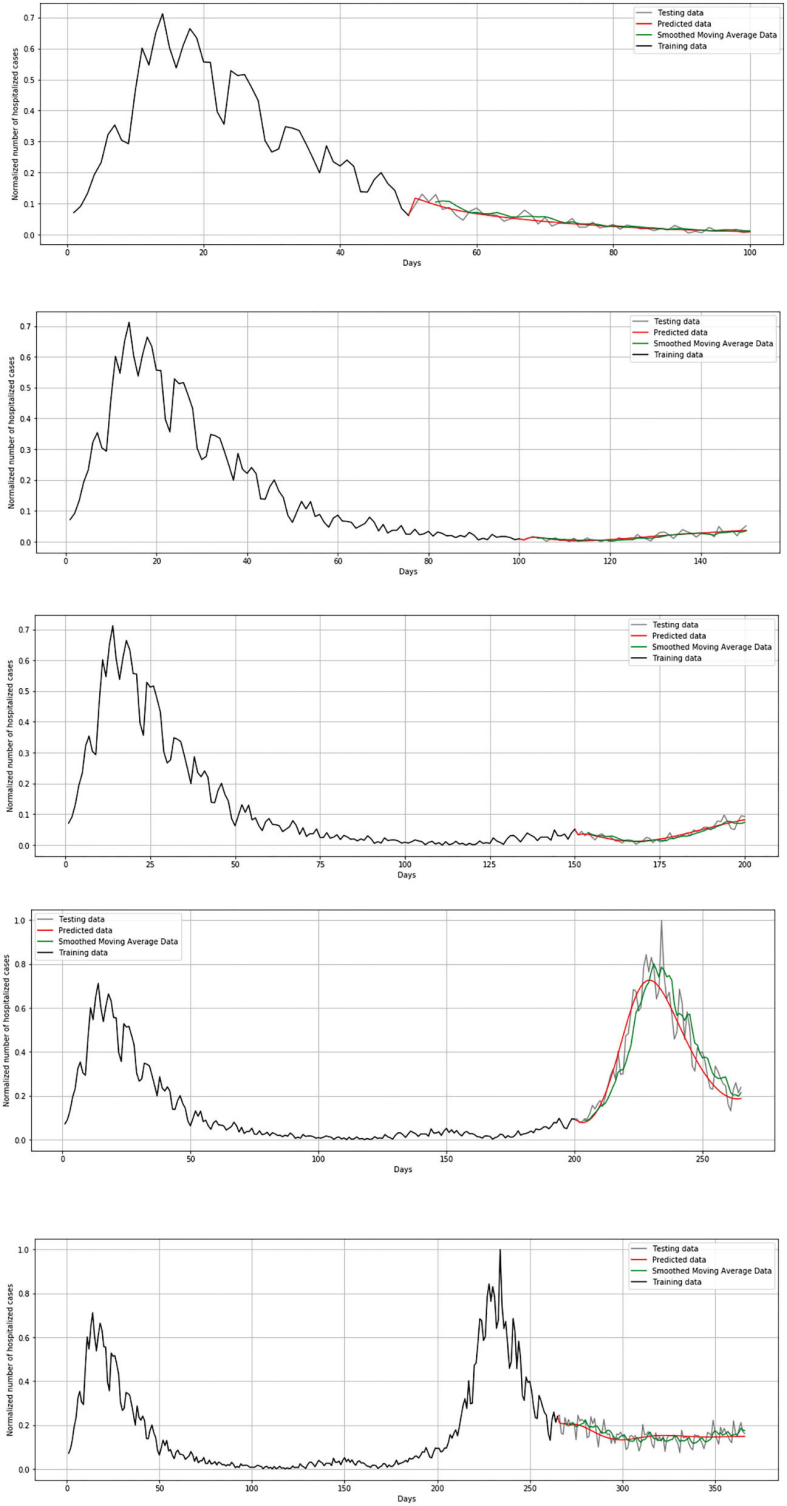
Cross-validation process for hospitalized cases in Belgium.

Unlike the mathematical model where the number of exposed, susceptible, infected, and deceased were simulated, for the LSTM-ED model, the focus is on univariate time-series data of daily infected, hospitalized (but not in ICU), patients in ICU, and deceased cases. We have used Tensorflow and Keras to implement the neural network in Python 3.7.4. The Keras function for the LSTM layer has an argument called the initial state that includes a list of initial state tensors to be transferred to the first cell call. We set this argument to the default value that involves zero-filled initial state tensors. We have adopted grid search method for hyperparameter optimization, and we iterate a different number of nodes that have been used in the hidden layer, dropout rate, optimizer, epochs, and batch size. The best number of neuron units in hidden layers is set to be 60, with a dropout rate of 0.4. As optimizer, Adam has been selected and batch size to be 32. The model has been trained during 100 epochs. We used the cross-validation process in order to get a more realistic view of the error and predictions of the model. It means that for the first iteration, we trained the LSTM-ED model with the 50-day dataset from March 15, 2020 to May 3, 2020, then we used the trained model to forecast each variable of the 50-day test dataset from May 4, 2020 to June 22, 2020. The mentioned process is repeated in additional four iterations. The second iteration implies training data from the beginning, March 15, 2020 to June 22, 2020, and the test data from June 23, 2020 to August 12, 2020. The third iteration implies training data up to August 12, 2020 and test data from August 13 2020 to October 2, 2020. It is clear that the training data in each subsequent case will include all the data that were analyzed in the previous iterations, including both the training and test sets of the previous iterations. The test dataset in the fourth iteration contains 62 samples instead of 50, and it means that data from October 3, 2020 to December 4, 2020 are included. The last iteration implies the training data from March 15, 2020 to December 4, 2020, and the test data from December 5, 2020 to March 15, 2021. In this case, the training set is larger than in previous iterations; accordingly, the test set is also larger and includes 100 days. The example of cross-validation process and mentioned iterations is presented in Figure 10. In the following example, the number of hospitalized cases in Belgium was used as input data.

**Figure 11:**
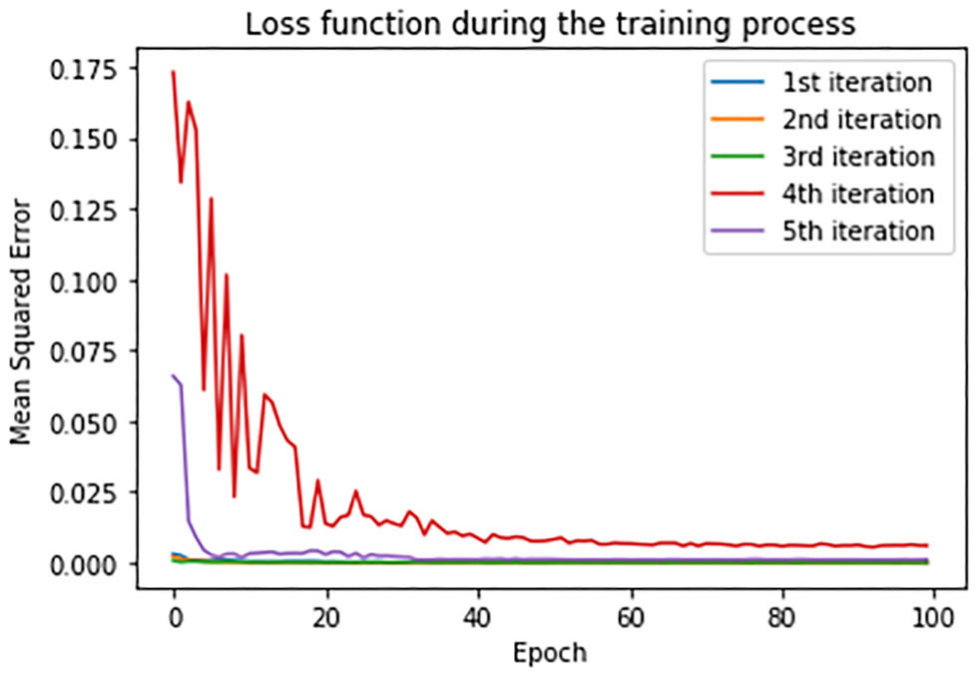
Loss function in training set during the cross-validation process.

In order to estimate the error between real and predicted values, mean squared error (MSE) has been set as a loss function. Loss function during the training process is shown in Figure 11. Loss function is presented in each iteration of the cross-validation process.

As evaluation metrics, root MSE (RMSE) mean absolute error (MAE) and R2 score are used. The average values of the metrics of all the five iterations for all the three countries is given in Table 5. Due to large fluctuations in the real data, we compared the forecasted curve with the smoothed version of the real curve. We applied the moving average smoothing technique to real values of the test dataset in order to remove the variation between time steps. Actually, we created a new series where the values comprised the average of 5 days of observations from the real data.

**Table 5:** The regression metrics on test data of Belgium, Netherlands and Luxembourg using LSTM model.

**Country**	**Belgium**			**Netherlands**			**Luxembourg**		
**Metrics**	**RMSE**	**MAE**	***R*^**2**^ score**	**RMSE**	**MAE**	***R*^**2**^ score**	**RMSE**	**MAE**	***R*^**2**^ score**
Infected	535.93	440.47	0.76	434.28	362.96	0.82	25	20.59	0.76
Severe (hospitalized)	20.42	17.16	0.83	94.6	79.43	0.78	12.24	10.45	0.83
Critical (ICU)	38.97	28.15	0.6	37.61	30.06	0.65	3.17	2.63	0.66
Deceased	8.72	7.54	0.73	5.23	4.18	0.66	0.38	0.31	0.77

**Figure 12:**
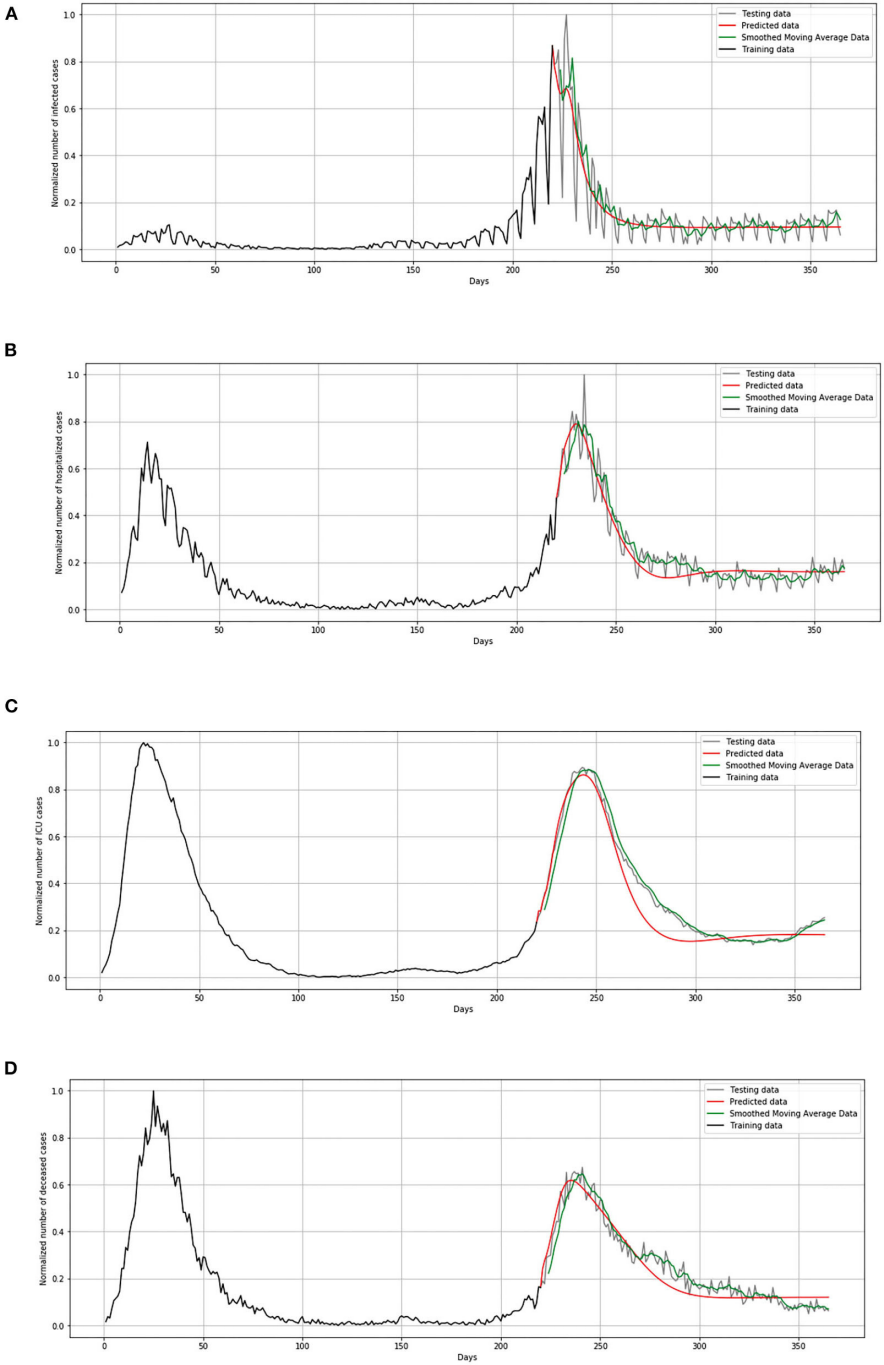
Comparison of real and simulated values for Belgium: **(A)** number of infected cases, **(B)** number of hospitalized cases, **(C)** number of ICU cases, **(D)** number of deceased cases.

During the validation process, we concluded that LSTM-ED is capable of predicting when another peak of the epidemic will occur, based on the position of the first peak. Therefore, we decided to show for each variable individually how the network will forecast the values for ~100 days. In the case of Belgium, in this 1-year period, only two peaks appeared, so the period October 20, 2020 to March 15, 2021 was used for the testing dataset. This means that the dataset is divided in the following manner: 58% for training and 42% for the testing process. The comparison between the official statistical data and simulated curves is shown in Figure 12. Also, a smoothed curve is presented in order to remove a noise and expose better a trend of the official data curve. The trend of the predicted curve matches well the smoothed curve from official data for all the monitored curves, infected, hospitalized, ICU, and deceased. For the case of Netherlands and Luxembourg, we made a different division and returned to the original decision to take a test set consisting of 100 days, which includes the data from December 6, 2020 to March 15, 2021.

**Figure 13:**
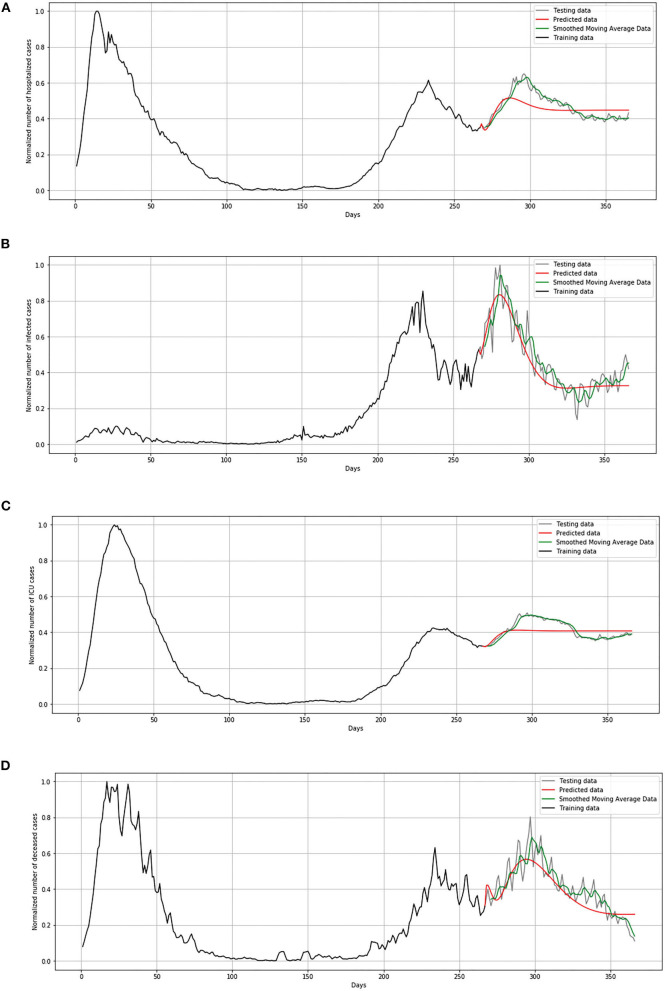
Comparison of real and simulated values for Netherlands: **(A)** number of infected cases, **(B)** number of hospitalized cases, **(C)** number of ICU cases, **(D)** number of deceased cases.

In Figure 13, comparison between the official statistical data of Netherlands and simulated curves is shown. It can be seen that in the cases of infected and deceased people, the trend is matching with very small deviations between official statistical and smoothed curves, as well as position of peaks. However, for the cases of hospitalized and ICU, the position of peaks is matching, but the height of the peaks differs a little more than in previous cases.

**Figure 14:**
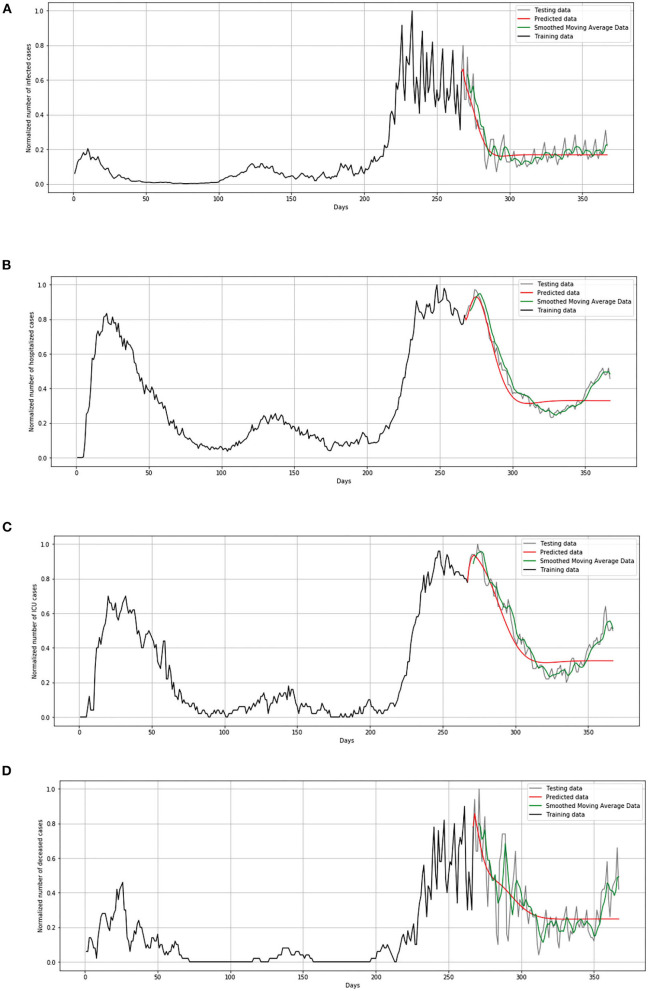
Comparison of real and simulated values for Luxembourg: **(A)** number of infected cases, **(B)** number of hospitalized cases, **(C)** number of ICU cases, **(D)** number of deceased cases.

In Figure 14, comparison between the official statistical data of Luxembourg and simulated curves is shown. The trend of the predicted curve matches well the smoothed curve from official data for all the monitored curves, infected, hospitalized, ICU, and deceased. However, in the last days of the test set, the simulated curve tends to flatten, so we can conclude that soon there will be no peak like the highest peak of the epidemic in Luxembourg.

The aim of our study was to establish the architecture of the model and hyperparameter settings in order to forecast several COVID-19 categories. Our model is able to forecast number of infected patients per day, number of hospitalized patients per day, number of patients in intensive care units, number of patients with a fatal outcome. As far as we know, most of the literature has been oriented toward predicting only the total or cumulative number of infected (positive) cases per day. Vadyala et al. (81) proposed K-means-LSTM neural network to construct a prediction model for short-term forecasting of confirmed COVID-19 cases in Louisiana, United States (81). As a result, the proposed method achieved forecasting performance with an RMSE value of 601.2. Ayyoubzadeh et al. (82) used LSTM and linear regression models to estimate the number of positive COVID-19 cases in Iran. To evaluate the robustness of the model, 10-fold cross-validation was utilized, and, as performance evaluation criterion, RMSE was used. According to the experimental results, the LSTM model achieved an RMSE value of 27.187 (82). Furthermore, Wang et al. (41) proposed a model based on LSTM neural networks in terms of daily and cumulative forecasting of infected patients in Russia, Peru, and Iran (41). They stated that the existing forecasting LSTM model can only predict the epidemic trend within the next 30 days accurately, so they included an additional mechanism for the purpose of long-term forecasting. Taking into account the limitations of previous studies, the aim from the aspect of deep learning methodology was to use an encoder-decoder LSTM model for long-term forecasting of the spread of COVID-infections in the Benelux Union. We select the countries with a reliable source of COVID-19 statistics, because in these countries the situation with and without social measures was present, with the aim to establish a model that will be able to forecast in a long-term manner. Selection of different countries as the test set does not affect the conceptual essence of this study, since the proposed methodology is general and can be applied with respect to the epidemic reality of any location and population. Our results show that the LSTM model proves to be promising for long-term forecasting, so the established methodology can be applied for any country or region.

### Complex Phenomena to Be Included as Possible Extensions of the Model

Modeling COVID-19 is very complex, and many phenomena are yet to be included in future models. Although the initial analyses from this study show promising results, some simplifications had to be adopted. We plan to address them in future:

- by the definition of the infected compartments, it is being assumed that asymptomatic cases are not infective. Although this is an idealized case, and many studies suggest that people infected with COVID-19 may be temporarily asymptomatic and infectious before developing symptoms, there are also studies that indicate that asymptomatic cases may not be infectious (83). Future expansion of the model will deal with infectious asymptomatic cases.
- stochastic formulation may be used to incorporate flexibility (uncertainty) in the predictions and obtain improved estimates of the parameters. In order to capture the stochastic nature of the transitions between the compartmental populations in such models, methods such as Markov Chain Monte Carlo can be used (84). Future discussion on improved model parameters will include the stochastic nature of epidemiological modeling.
- although LSTMs have proven efficient in terms of utilizing time-series data for prediction, there is a gap in the existing solutions that can combine both spatial and temporal aspects of the dynamics of COVID-19. Melin et al. proposed self-organizing neural networks and a fuzzy fractal approach in order to bridge this gap and address the issue of spatial variation (85). Although their results seem promising, the validation of results is only shown on 10 days prediction, which does not guarantee the accurate prediction of longer periods. Khan et al. (86) proposed a hybrid convolutional neural network (CNN) and long short-term memory (LSTM) model in order to extract multi time-scale features from convolutional layers of CNN and to learn short, medium, and long time series dependencies (86). Their approach seems promising, with results reported to be better in comparison with LSTM networks. This is also the direction we will investigate in the future.
- mobility-contact effects were not taken into account at this point of model validation. During the first week of new measures (starting on Friday, March 13) a steep drop in mobility between many cities has been noted. Mobility in March decreased to around 50%, 60% of January levels (87). From the perspective of neighboring countries, the border cities in Germany and in the Netherlands also noted less traffic, but the drop happened a little bit slower than in Belgium. In the border cities of Lille and Luxembourg, traffic is significantly reduced, at the highest level, among the considered regions. Furthermore, even though Dutch and German border cities noticed slightly higher inner-city mobility than the cities in Belgium, the drop in cross-border traffic decreased to around 50%, 60% of January level of traffic. If we consider Belgian inner-city traffic, the last week of March was characterized by an even more drastic reduction in overall mobility hovering just about 30% of January levels. Traffic between cities dropped between 10 and 20% of their January levels. If we take into account these statistics and information that Belgium tightened measures after March 17 and introduced complete lockdown, this can imply that cross-border mobility might not have a significant impact on the spread of the virus and worsening of the current situation in these countries. Different methods can be used to improve the validation and verification processes in order to find the parameters of the system dynamics model, meaning the parameters of the pandemic (88) discussed that in their study the effect of lockdowns can be tracked by a reduction in mobility reported by Google (89). In this study, we are focused on describing how the comparison between the standard SEIRD model and the LSTM model has achieved high accuracy results.

The SARS-CoV-2 virus already has hundreds of variations. Pathogen surveillance is performed for each country by the national authority. Although first identified on April 6 2021 in Belgium, the delta (B.1.617.2) variant of concern in August 2021 is dominant lineage in the country, accounting for more than 95% of all the variants (90). According to preliminary findings, this variation is also related with greater transmissibility and quicker spread. For Netherlands, RIVM, The National Institute for Public Health and the Environment is undertaking laboratory research to determine which versions of the virus are present and what this means for the spread of the virus in the country. Out of the total number of samples, the most frequent variant is the alpha (B.1.1.7) variant, after which the delta (B.1.617.2) and beta (B.1.351) versions are less frequent (91). All the three variants have had an estimated reproduction number higher than that of the old variant of the virus. In Luxembourg, community surveillance showed that the delta (B.1.617.2) variant represents the dominant one (accounting for 99.1%), with low prevalence of the gamma (P.1) variant (accounting for 0.9%) (92). This major question of variants will help in terms of investigation whether they are more easily spread, cause more illness, or if viral variations do not react as well to immunization.

Additionally, at this point, there are very little publicly available epidemiological data per day on patients with COVID-19 with respect to variants, especially on the level of the country. Results from genome-wide association studies (GWAS) in terms of trait-associated genetic variants can be used as control variables in epidemiology studies to account for confounding genetic group differences (93). The harmonized individual-level data of some participating cohorts from Belgium (BeLCovid_2), Brazil (BRACOVID), Italy (COVID19-Host(a)ge_4, GEN-COVID), Spain (COVID19-Host(a)ge_1,2,3, INMUNGEN-CoV2, SPGRX), and Sweden (SweCovid) are under preparation to be deposited at the European Genome-phenome Archive (EGA) (94). This will be a future direction for research and model update.

## Conclusions

This study describes the modeling of COVID-19 spread and development in the population, using two proposed methodologies, the SEIRD model and the model based on LSTM neural networks. The COVID-19 epidemic was declared a pandemic by the WHO since the number of infected people grows exponentially, and many countries have decided to impose certain measures, such as a complete lockdown of affected cities to reduce the number of contacts and stop the spread of the virus. Our proposed method included the SEIRD compartmental epidemiological model with included components, susceptible, exposed, infected (we have divided the infected group into three subgroups, mild, severe, and critical), recovered, and deceased, with included effects of lockdown modeling. In order to calculate the parameters for the model, we have also investigated official statistical data for the countries of Benelux (Belgium, Netherlands, and Luxembourg). The results show that the SEIRD model is able to accurately predict several peaks for all the three countries, and increase and decrease in the number of infected people, with only higher RMSE for mild cases. On the other hand, the second proposed method, LSTM networks show that they are capable to predict later peaks based on the position of previous peaks with the low values of RMSE. Higher values of RMSE are observed in the forecasting of daily infected cases due the thousands of infected people per day in those countries. The match between simulated and real values can be affected by several things, such as underreporting of the number of cases, estimating initial conditions, and setting parameters. In general, if we take into account all the three countries, official and simulated values show a good match, which means that the model is showing promising results and can be further upgraded to take into account different underlying complex phenomena. Future research will include more phenomena, especially medical intervention and asymptomatic infection, mobility of people, population density, economic and social aspects, variants of concern, etc., in order to better describe the spread and development of COVID-19. We will also test the model on a greater number of countries.

## Data Availability Statement

The original contributions presented in the study are included in the article/supplementary material, further inquiries can be directed to the corresponding authors.

## Author Contributions

TŠ, AB, and NF implemented the algorithms for epidemiological modeling. DC and AC collected the official data from websites. IL, SŠ, and ZC provided critical feedback and helped shape the research, analysis, and manuscript. DM and DB provided valuable discussion in the analysis of the obtained results and upgrading of the model. Article concept was created by NF and ZC, drafted by TŠ and AB, and written by all the authors. All the authors discussed the results and contributed to the final manuscript.

## Funding

This research was funded by Serbian Ministry of Education, Science and Technological Development [451-03-68/2020-14/200107 (Faculty of Engineering, University of Kragujevac) and 451-03-68/2020-14/200378 (University of Kragujevac, Institute for Information Technologies, Kragujevac)]. This research was also supported by the CEI project Use of Regressive Artificial Intelligence (AI) and Machine Learning (ML) Methods in Modelling of COVID-19 spread–COVIDAi.

## Conflict of Interest

The authors declare that the research was conducted in the absence of any commercial or financial relationships that could be construed as a potential conflict of interest.

## Publisher's Note

All claims expressed in this article are solely those of the authors and do not necessarily represent those of their affiliated organizations, or those of the publisher, the editors and the reviewers. Any product that may be evaluated in this article, or claim that may be made by its manufacturer, is not guaranteed or endorsed by the publisher.
